# Improving end-of-life care in acute geriatric hospital wards using the Care Programme for the Last Days of Life: study protocol for a phase 3 cluster randomized controlled trial

**DOI:** 10.1186/s12877-015-0010-7

**Published:** 2015-02-22

**Authors:** Rebecca Verhofstede, Tinne Smets, Joachim Cohen, Massimo Costantini, Nele Van Den Noortgate, Luc Deliens

**Affiliations:** End-of-Life Care Research Group, Vrije Universiteit Brussel (VUB) & Ghent University, Brussels, Belgium; Palliative Care Unit, IRCCS Arcispedale S. Maria Nuova, Reggio Emilia, Italy; Department of Geriatrics, Ghent University Hospital, Ghent, Belgium; Department of Medical Oncology, Ghent University Hospital, Ghent, Belgium

**Keywords:** Cluster randomized controlled trial, Terminal care, Hospital, Older people

## Abstract

**Background:**

The Care Programme for the Last Days of Life has been developed to improve the quality of end-of-life care in acute geriatric hospital wards. The programme is based on existing end-of-life care programmes but modeled to the acute geriatric care setting. There is a lack of evidence of the effectiveness of end-of-life care programmes and the effects that may be achieved in patients dying in an acute geriatric hospital setting are unknown. The aim of this paper is to describe the research protocol of a cluster randomized controlled trial to evaluate the effects of the Care Programme for the Last Days of Life.

**Methods and design:**

A cluster randomized controlled trial will be conducted. Ten hospitals with one or more acute geriatric wards will conduct a one-year baseline assessment during which care will be provided as usual. For each patient dying in the ward, a questionnaire will be filled in by a nurse, a physician and a family carer. At the end of the baseline assessment hospitals will be randomized to receive intervention (implementation of the Care Programme) or no intervention. Subsequently, the Care Programme will be implemented in the intervention hospitals over a six-month period. A one-year post-intervention assessment will be performed immediately after the baseline assessment in the control hospitals and after the implementation period in the intervention hospitals. Primary outcomes are symptom frequency and symptom burden of patients in the last 48 hours of life.

**Discussion:**

This will be the first cluster randomized controlled trial to evaluate the effect of the Care Programme for the Last Days of Life for the acute geriatric hospital setting. The results will enable us to evaluate whether implementation of the Care Programme has positive effects on end-of-life care during the last days of life in this patient population and which components of the Care Programme contribute to improving the quality of end-of-life care.

**Trial registration:**

ClinicalTrials.gov Identifier: NCT01890239. Registered June 24th, 2013.

## Background

Pain and symptom management, appropriate treatments and medication and communication about end-of-life issues are identified as key elements of quality care for terminally ill patients [1]. However, clinicians are often inadequately prepared to diagnose dying effectively [2] or to discuss the likelihood of imminent death with patients and families [3-7]. Studies have also shown that older hospitalized people are less likely to receive appropriate pain control and more likely to receive burdensome interventions at the end of life than their younger counterparts [8-11]. Although end-of-life care has been identified as a priority for older people [8,12] and a large proportion die in hospital [13-15], the quality of end-of-life care for older hospitalized patients is suboptimal, leaving room for improvement [16]. As a significant number of older patients may die within the acute geriatric ward of a hospital, it is an important setting in which end-of-life care could be improved.

**Figure 1:**
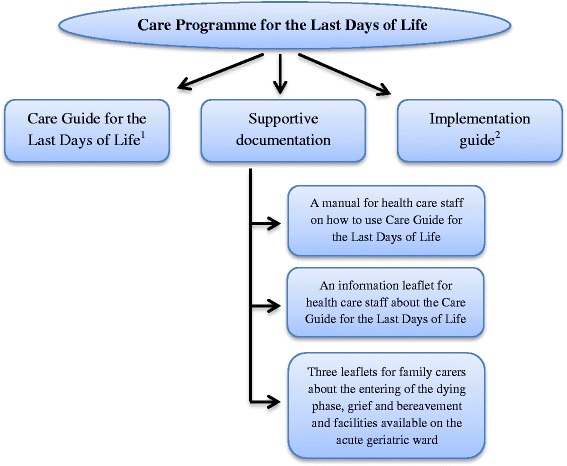
**The Care programme for the last days of life.** ^1^A multi-professional document that provides a template of care for the last days and hours of life with recommendations on different aspects of care and guidance for the psychological and spiritual support of patients and their families. ^2^This guide assists health care staff in implementing the Care Programme for the Last Days of Life on the geriatric ward during a six-month period.

To improve the quality of care at the end of life in the geriatric hospital population we developed and successfully piloted the Care Programme for the Last Days of Life (Verhofstede R, Smets T, Cohen J, Costantini M, Van Den Noortgate N, van der Heide A, Deliens L: Development of the care programme for the last days of life for older patients in acute geriatric hospital wards: a phase 0–1 study according to the Medical Research Council Framework, submitted; Verhofstede R, Smets T, Cohen J, Costantini M, Van Den Noortgate N, Deliens L: Feasibility and preliminary effects of the Care Programme for the Last Days of Life in an older acute hospital population: mixed-methods study of the success of implementation and staff perceptions, in preparation). This programme is based on the Liverpool Care Pathway (LCP) programme, taking into account the concerns regarding the LCP raised in the UK and adapted to the geriatric hospital population and setting (Verhofstede R, Smets T, Cohen J, Costantini M, Van Den Noortgate N, van der Heide A, Deliens L: Development of the care programme for the last days of life for older patients in acute geriatric hospital wards: a phase 0–1 study according to the Medical Research Council Framework, submitted). The Care Programme essentially aims to raise awareness among geriatric health care staff of the importance for improving end-of-life care and to prepare them for a change in end-of-life care, to train staff in delivering good end-of-life care with the support of a multi-professional document called the Care Guide for the Last Days of Life, to support dying geriatric patients with the Care Guide for the Last days of Life, to regularly evaluate the delivered end-of-life care and support and to further educate the staff in delivering optimal end-of-life care. The Care Programme consists of the following documents: (1) the Care Guide for the Last Days of Life, (2) supportive documentation and (3) an implementation guide (Figure 1).

Although end-of-life care programmes have been developed since the 1990s and have already been implemented in more than 20 countries [17], the available evidence regarding their effectiveness is weak and studies are limited to the cancer population. Two systematic reviews conclude that randomized controlled trials or other well designed controlled studies are needed to obtain additional evidence about the effectiveness of end-of-life care pathways [18]. To date, only one cluster randomized controlled trial has been performed to study the effects of the LCP in oncology patients dying in Italian hospitals [19,20]. Results of that study show that a well-implemented LCP programme has the potential to reduce the gap in quality of care between hospices and hospitals. However, the results also show that the effects of the LCP programme are smaller than those shown in qualitative and before-and-after non-controlled studies, and no significant effects on the overall quality of care were found [20]. Nonetheless, the effectiveness of the LCP programme was evaluated in a cancer population whereas end-of-life care pathways or programmes are often used for patients who are dying from diseases other than cancer. Furthermore, the study was underpowered and therefore may have led to underestimated results.

Although it is now widely accepted that clinical practice should be, wherever possible, evidence-based, clinical pathways to improve the quality of end-of-life care are often implemented without a thorough evaluation of their effectiveness [21]. Additional and robust evidence is required [18,22] before realizing a large scale implementation of an end-of-life care pathway. Hence, a thorough evaluation of the effectiveness of the Care Programme for the Last Days of Life is needed before implementing it in practice. We will therefore evaluate the Care Programme in a phase 3 trial according to the MRC framework [23].

The aim of this article is to describe the research protocol of the cluster randomized controlled trial that will be performed to evaluate the effectiveness of a complex intervention, the Care Programme for the Last Days of Life, in acute geriatric wards.

## Methods

### Trial design

**Figure 2:**
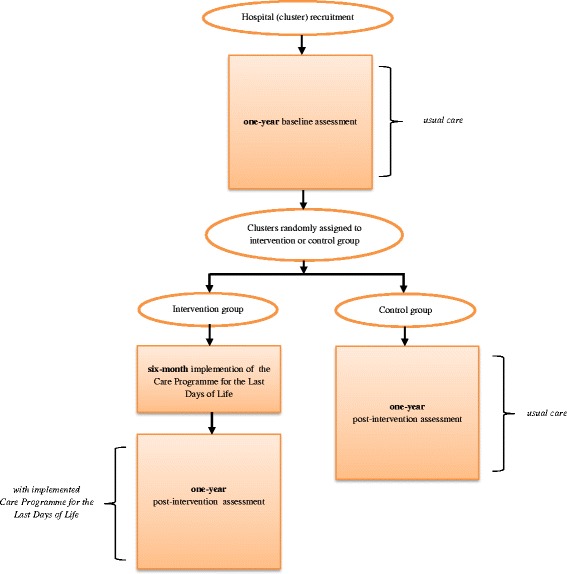
**Flowchart of the cluster randomized controlled trial.**

While a classic randomized clinical trial is known as the most appropriate method to study the effect of an intervention, it is impossible to randomize a complex intervention within a hospital without contamination of the control arm [24]. For this reason, a multicentre two arm cluster randomized controlled trial will be performed. To prevent possible bias at the level of the hospital, a clustering will take place on the hospital level. Consequently, randomization will be carried out at the level of the hospital. The flow diagram of the study protocol is outlined in Figure 2. The CONSORT guidelines have been followed to design this study [25]. The trial is registered in ClinicalTrials.gov, identifier NCT01890239.

### Study population

The inclusion criteria of the hospitals in the trial are:

- ○ the cluster or hospital has one or more acute geriatric wards
- ○ the medical and nursing head of one or more acute geriatric wards per hospital give consent for participation in the study

The inclusion criteria of patients are:

- ○ those dying in the acute geriatric ward between October 2012 and March 2015
- ○ those that having been hospitalized for more than 48 hours
- ○ those having given informed consent at admission for the use of their personal information from medical or nursing records for the purposes of the study.

### General procedures of the cluster RCT

First, a one-year baseline assessment will be conducted in all participating acute geriatric wards of participating hospitals. During that period care will be provided as usual. At the end of the baseline assessment all participating hospitals with one or more participating wards will be randomized into intervention or control groups. In the intervention group the Care Programme for the Last Days of Life will be implemented over a six-month period with the support of an implementation guide (Figure 2). After the implementation period, the intervention group will conduct a one-year post-intervention assessment during which the Care Guide for the Last Days of Life will continue to be used. The control group will continue to provide care as usual and will conduct a one-year post-intervention assessment directly following the one-year baseline assessment (Figure 2).

### Intervention

The Care Programme aims to introduce and embed the Care Guide for the Last Days of Life, which will be initiated when a patient is diagnosed as dying and which provides a comprehensive template of evidence-based, multidisciplinary care for the last days and hours of life. The Care Programme will be implemented and subsequently established according to an implemention guide incorporating nine components: (1) establishing the implementation project and preparing the environment for organizational changes, (2) preparing the documentation, (3) baseline review, (4) training geriatric health care staff, (5) use of the Care Guide for the Last Days of Life with intensive support, (6) semi-intensive support, (7) evaluation, (8) consolidation and (9) ongoing education, training and support (Table 1).

**Table 1:** **Overview of the nine components within the implementation guide**

**N°**	**Content**
**Component 1**	**Establishing the implementation project and preparing the environment**
	▪ Informing the geriatric health care staff about the implementation project and the importance of change in care during the last days of life
	▪ Executive endorsement: acquiring management approval for the trainings and audits
	▪ Involvement of specialist palliative care services is recommended: at least one member of the Palliative Support Team of the hospital is member of the steering group
	▪ Facilitators: a nurse and a physician of the geriatric ward
	▪ Formation of steering group: at least four people from the geriatric ward (facilitators included)
	▪ Intensive 2-day training of facilitators
**Component 2**	**Preparing the documentation**
	▪ Development of an information leaflet for family carers about the facilities in the geriatric hospital ward
**Component 3**	**Baseline review**
	▪ Analyzing end-of-life care data of deceased geriatric hospital patients using the patients’ medical files
**Component 4**	**Training geriatric health care staff**
	▪ Feedback of the results to the staff and focusing on improvement within the geriatric ward
	▪ Facilitators and specialist palliative care colleagues train geriatric health care staff with the aid of a training package (i.e. hand-outs with information about the Care Guide for the Last Days of Life, a copy of the Care Guide for the Last Days of Life, a casus to discuss in group etc.)
**Component 5**	**Care Guide use and intensive support**
	▪ Care Guide use after sufficient training and education
	▪ Intensive support and supervision by the steering group through repeated coaching, telephone and direct guidance, discussion of clinical cases and clinical audits
**Component 6**	**Semi-intensive support**
	▪ Semi-intensive support and supervision by the steering group through repeated coaching, telephone and direct guidance, discussion of clinical cases and clinical audits
**Component 7**	**Evaluation**
	▪ To organize a qualitative evaluation of the implementation: evaluating and discussing the performance and progress of each of the previous components
	▪ The qualitative evaluation acknowledges areas where further support, education or training is needed
**Component 8**	**Consolidation**
	▪ To adopt a strategy to maintain/improve the implementation and sustainability of the Care Guide
	▪ Support and supervision by the steering group through repeated coaching, telephone and direct guidance, discussion of clinical cases and clinical audits
**Component 9**	**Ongoing education, training and support**
	▪ Keeping up to date with developments in end-of-life care and a continuing education and evaluation within the hospital ward

The development and content of the Care Programme for the Last Days of Life are extensively described elsewhere (Verhofstede R, Smets T, Cohen J, Costantini M, Van Den Noortgate N, van der Heide A, Deliens L: Development of the care programme for the last days of life for older patients in acute geriatric hospital wards: a phase 0–1 study according to the Medical Research Council Framework, submitted).

### Outcome measures

#### Primary outcome

Quality of dying during the last 48 hours of life: the patient’s symptom frequency and symptom burden measured using the EOLD-SM and EOLD-CAD [26].

#### Secondary outcomes

1. the quality of care during the last three days of life as perceived by nurses, i.e. physical symptoms, emotional, psychological and spiritual/existential needs and provision of information and support measured using the POS [27]
2. the quality of care during the last 48 hours of life as perceived by family carers, i.e. satisfaction with the care provided to the patient during the last 48 hours of life measured using the EOLD-SWC [26]
3. the content of care during the last 48 hours of life, i.e. the goal of treatment, medical and nursing interventions, medication policy
4. the communication among clinical staff, i.e. informing the family physician about the impending death
5. the communication between clinical staff and patients and/or family carers, i.e. the perception of communication with the physician during the dying phase by family carers measured using the FPPFC [28]
6. the level of bereavement of family carers after the death of the patient measured using the PGD scale [29].

#### Process evaluation

We will also evaluate the quality of the process of implementation in the intervention group. An evaluation tool was developed to measure the degree to which the Care Programme for the Last days of Life was implemented in each ward in compliance with the implementation guide. For each component of the Care Programme indicators were developed so that the implementation of each individual component as well as the entire implementation process could be documented and quantitatively evaluated. This evaluation tool has been developed and piloted in a phase 2 study (Verhofstede R, Smets T, Cohen J, Costantini M, Van Den Noortgate N, Deliens L: Feasibility and preliminary effects of the Care Programme for the Last Days of Life in an older acute hospital population: mixed-methods study of the success of implementation and staff perceptions, in preparation).

### Measurement instruments

Primary and secondary outcomes will be measured retrospectively after each death on the ward using questionnaires to be filled out by three different respondent types: the nurse who was most closely involved in the care for the deceased patient, the physician who was most closely involved in the care for the deceased patient, and a family carer of the deceased patient. The nurse and family carer questionnaires contain validated measurement instruments, in addition to self-developed questions. The physician questionnaire only contains self-developed questions (Table 2). Regarding the validated measurement instruments, the nurse questionnaire contains: the End-of-Life in Dementia Scales Symptom Management (EOLD-SM) [26], the End-of-Life in Dementia Scales Comfort Assessment in Dying Management (EOLD-CAD) [26] and the Palliative care Outcome Scale (POS) [27].

**Table 2:** **Content of the three different questionnaires for nurses, physicians and family carers**

**Questionnaire**	**Questions**	**Scale**	**Primary and secondary outcomes**
**Nurse**	Used from a scale	EOLD-SM	Symptom frequency*
		EOLD-CAD	Symptom burden*
		POS	Quality of care
	Self-developed questions		Content of care, i.e. nursing interventions,
			Communication between clinical staff and patients and/or family carers of dying patients
			Communication among clinical staff
**Physician**	Self-developed questions		Content of care, i.e. goal of treatment, medical interventions, medication policy
			Communication among clinical staff
**Family carer**	Used from a scale	EOLD-SM	Symptom frequency*
		EOLD-CAD	Symptom burden*
		FPPFC	Communication between clinical staff and patients and/or family carers of dying patients
		EOLD-SWC	Quality of care
		PGD	Level of bereavement
	Self-developed questions		Communication between clinical staff and patients and/or family carers of dying patients

*primary outcome.

In the questionnaire for the family carer the following validated instruments are included: the EOLD-SM [26], the EOLD-CAD [26], the End-of-Life in Dementia Scales Satisfaction With Care (EOLD-SWC) [26], the Family Perception of Physician-Family Caregiver Communication (FPPFC) [28] and the Prolonged Grief Disorder (PGD) Scale [29]. All three questionnaires have been cognitively tested in face-to-face interviews with four nurses, four physicians and three family carers respectively, and were subsequently refined where needed.

### Data collection

During the baseline and post-intervention assessment, questionnaires will be filled in for all patients who died in the participating geriatric hospital wards and who met the inclusion criteria. The nurse and physician most closely involved in the care of the deceased patient will be asked to fill in a questionnaire within one week of the death. Six weeks after the death the researcher will send a questionnaire to a family carer, if they have given informed consent to being contacted by the researcher. In cases where the family carer does not respond to the questionnaire up to two reminders will be sent, two weeks after the initial sending of the questionnaire and two weeks later.

### Sample size calculation

The hypothesis of this cluster randomized trial is that there will be significant differences in symptom frequency and symptom burden between patients dying in the intervention group and those dying in the control group. Symptom frequency and symptom burden will be measured using the EOLD-SM and the EOLD-CAD. Because our primary outcome is a reduction in symptom frequency and symptom burden during the last 48 hours of life, we consider a total EOLD-CAD score of 3.2 (7.6%) as the minimum clinically important difference for implementing the Care Programme for the Last Days of Life [30]. A minimum increase of 3.2 in the intervention group compared to the control group corresponds with an effect size (EC) of about 0.55. A minimum change of 5% to 10% has been found to be clinically significant for symptom and quality-of-life analyses [31]. In order to calculate the sample size of this cluster trial two other elements are essential: the intra-cluster correlation coefficient and the average size of the cluster (number of cluster deaths). We estimate an intra-cluster correlation coefficient of between 0.02 and 0.05 [32] and a conservative average of 30 deaths per hospital per year based on observed mortality statistics, taking into consideration the non-included deceased patients. In Table 3 four ICC scenarios (from 0.02 to 0.05) intersect with three scenarios of average size of the cluster (from 20 to 40). This table reports different sample size scenarios necessary to detect an ES of 0.55 with alpha = 0.05 and a power of 80% conditional on the hypothesized levels of ICC (from 0.02 to 0.05) and average size of cluster (from 20 to 40).

**Table 3:** **Total number of clusters required according to different average sizes of the clusters and ICC (ES = 0.55, alpha = 0.05, power = 0.80)**

		**Average size of the clusters**		
		20	30	40
**ICC**	0.02	10	8	8
	0.03	12	10	10
	0.04	12	10	10
	0.05	14	*12*	10

A sample size of six clusters per group with 30 individuals per cluster achieves 80% power to detect a difference of 3.2 between the group means when the intracluster correlation is 0.05 using a Two-Sided *T*-test with a significance level of 0.05.

### Recruitment of hospitals

Based on the cluster size calculation, 10 to 12 hospitals with one or more acute geriatric wards must be recruited. In order to recruit these hospitals, the study has been presented at three geriatric meetings. Shortly after these meetings information letters were sent to geriatricians. Geriatricians who were interested in participating were contacted by the researcher to make an appointment to explain and discuss the study and to sign an agreement of participation form. If geriatricians did not spontaneously contact the researcher, the researcher took the initiative herself to contact the geriatricians by phone about their interest and possible participation.

### Randomization

At the end of the baseline assessment the included hospitals with one or more participating acute geriatric wards will be randomly assigned to the intervention group (implementing the Care Programme for the Last Days of Life) or to the control group (usual care).

As the number of clusters to be randomized is considerably smaller than in trials where the unit of randomization is the patient, there is a chance of baseline imbalance between the randomized groups. The risk of baseline differences can be reduced using pair-matched randomization [33]. Hospitals will be matched in comparable pairs in terms of 1) the number of deaths per year for the participating geriatric wards, and 2) the motivation of the participating wards in terms of the number of patients from whom they will acquire informed consent for participation in the baseline measurement period. Information related to the number of deaths and motivation per hospital will be sent to a statistician outside the research group, who will then match the pairs and randomize the hospitals into the experimental and control group using a random number generator.

### Statistical analysis

All data collected through the three different questionnaires will be stored and collected in Ghent University Hospital using IBM SPSS Statistics. Data cleaning will be performed via SPSS syntax operations. All statistical tests will be done two-tailed with 95% confidence intervals. A p-value <0.05 will be considered statistically significant.

#### Descriptive statistics

Cluster and patient characteristics will be reported as mean and standard deviation (SD) or frequency and percentage respectively for continuous and categorical variables. The distribution of characteristics of clusters allocated as experimental or control hospitals will be compared with the Student *t*-test (for continuous variables), with non-parametric tests (for ordinal variables) and with the Pearson Chi-square (for binary or nominal variables).

#### Multivariable analysis

Our primary aim is to detect any differences in the EOLD-SM [26] and EOLD-CAD [26] between those dying in the intervention wards and those dying in the control wards. The primary statistical analysis will be by intention-to-treat, using multi-level models, taking into account clustering by hospitals. Because these primary outcomes are continuous, hierarchical linear models will be used which will be adjusted for the average level of quality of life and quality of care provided to the baseline assessment. This method of analysis will also be used for our secondary outcome measures. For the assessment of categorical secondary outcomes, the hierarchical logistical model will be used.

### Informed consent procedure

In order to guarantee privacy for patients whose data is collected in the study, certain procedures are necessary. As the Central Ethics Committee requires that data can only be collected from deceased patients who have given informed consent prior to the study, written informed consent to use personal data for the study will be requested by a nurse from each patient at the time of admission of the patient to the ward. If the patient is lacking in capacity, written informed consent will be requested from a family carer. Questionnaires will be filled in only for patients with informed consent at admission.

The physician and the nurse who were closely involved in the care of a deceased patient will be asked to fill in a questionnaire about the patient. If one of them refuses to complete the questionnaire the nurse who is responsible for the study on the ward will pass the questionnaire to another nurse or physician. A closely involved family carer will also be asked to fill in a questionnaire about their deceased relative and about their own experiences of care in the dying phase. In order for the researcher to be allowed to send a questionnaire to the family carer, a nurse will ask informed consent of the family carer shortly after the death of the patient. Family carers who give informed consent will be asked to sign a written informed consent form including their contact details. If the nurse in the hospital is unable to ask informed consent from the family carer shortly after the death of the patient, the hospital will send an informed consent form by post to the family carer two weeks after the death of the patient asking permission for the researcher to contact them. Family carers who give informed consent to being contacted by the researcher will be sent a questionnaire six weeks after the patient’s death. Family carers are also asked for their informed consent to fill in the questionnaire.

### Ethical approval

The study is approved by the Central Ethics Committee of the Vrije Universiteit Brussel (VUB) (Belgium) and by the Local Ethics Committees of the participating hospitals in Flanders.

## Discussion

This will be the first cluster randomized controlled trial to evaluate the effectiveness of the Care Programme for the Last Days of Life for the acute geriatric hospital setting. Following a baseline assessment, geriatric hospital wards will be randomized to the intervention or control group where the Care Programme will be implemented or care will be provided as usual. A post-intervention assessment should allow us to detect differences in the symptom frequency and symptom burden between patients in the intervention wards and those in the control wards.

A cluster RCT design has several important strengths. The first advantage of this robust design is that a control group will be used. Working with control hospitals can avoid the situation where differences between the baseline and post- intervention assessments within the intervention group are caused by changes other than the intervention that is being studied. Secondly, in all participating hospital wards the quality of care and the quality of life during the last 48 hours of life will be assessed before and after implementation of the care programme. That means that we will be able to compare end-of-life care in geriatric hospital wards before and after the implementation of our intervention, and that each hospital operates as its own control. A third strength of this design is that cluster randomized trials, unlike individually randomized controlled trials, can reduce the effect of treatment contamination as one or more geriatric wards from one hospital will handle the same care principles [34]. Fourth, this design may also increase compliance due to group participation.

This study also has several limitations. Firstly, the study questionnaires address symptoms and care during the last 48 hours of life, which is more or less the target period of the Care Guide. However, it is unknown how long the geriatric patients will be supported by the Care Guide for the Last Days of Life. Earlier studies have shown that the median duration and average time of use of the LCP in the hospital setting was 16 and 29 hours respectively [35,36]. However, another study found that 44% of hospice patients were supported by the LCP during two days [37]. We therefore cannot preclude that we may be measuring the quality of care during a period when the Care Guide had not yet been put into effect, which could dilute the apparent effect of the Care Programme. A second limitation, inherent to the focus on the last 48 hours of life, is that evaluations of a patient’s quality of life, content of care and communication will depend on after-death evaluations by proxies (nurses, physicians and family carers). However, in selecting the items for the questionnaires, we have taken into account the reliability and validity of proxy-reporting. Proxy measurements have, for instances, been shown to be relatively valid for relatively objective information such as the processes of care [38]. In former retrospective studies, bereaved family carers and professional caregivers, like nurses and physicians, have acted as proxy respondents and the reliability of proxy assessments for various aspects of end-of-life care and quality of life are well described [39]. The aspects we choose to measure are those that have shown sufficient agreement between patients and proxy respondents: observable physical symptoms, evaluation of care, service use and awareness of diagnosis [40]. We will also investigate some aspects that are more subjective such as psychological symptoms. It is known that in comparison with patients, nurses and family carers tend to overestimate the severity of such symptoms whereas physicians tend to underestimate them [39]. We therefore take into account the different perspectives of nurses, physicians and family carers.

Most end-of-life care pathways or programmes such as the LCP have been studied in different healthcare settings and have focused mainly on oncology patients. However, due to the ageing population and a simultaneous increase in the incidence of chronic diseases, future research evaluating the effects of end-of-life care pathways or programmes should also focus on elderly people dying of causes other than cancer [12,8]. Evaluating the Care Programme for the Last Days of Life for the acute geriatric hospital patient would therefore add evidence of the effectiveness of initiatives aimed at improving the quality of life and care for older patients dying in acute geriatric hospital wards.

By using a cluster randomized controlled trial design, the proposed study will contribute substantially to the increase in evidence for end-of-life care interventions. To our knowledge only one other cluster RCT in this area has studied the effects of the LCP programme on cancer patients dying in Italian hospitals [19,20]. A cluster RCT is a challenging, high-risk research design. However, results from a before-after cluster phase 2 trial support the need for multi-centre cluster randomized controlled trials [41], as this is the only feasible method of assessing the effectiveness of end-of-life care interventions [19].

## Conclusions

This will be the first cluster randomized controlled trial aimed at evaluating the effectiveness of the Care Programme for the Last Days of Life to improve the quality of care and quality of life during the last 48 hours of life of patients dying in acute geriatric hospital wards. Using this robust study design will allow us to describe in detail the quality of care and quality of life of elderly people dying in hospitals and will add to the evidence about the effectiveness of the Care Programme in the acute geriatric hospital setting. The poor quality of end-of-life care in hospitals remains a concern and dealing with that problem is a public health priority. We hope that this study will not only show whether the Care Programme for the Last Days of Life is effective in geriatric hospital wards but will also provide an understanding of the contribution of the different components of the Care Programme to end-of life care.

## Abbreviations

MRC Framework: Medical Research Council Framework
LCP: Liverpool Care Pathway
CONSORT: Consolidated standards of reporting trials
Cluster RCT: Cluster Randomized Controlled Trial
EOLD-SM: End-of-Life in Dementia Scales Symptom Management
EOLD-CAD: End-of-Life in Dementia Scales Comfort Assessment in Dying Management
EOLD-SWC: End-of-Life in Dementia Scales Satisfaction With Care
FPPFC: Family Perception of Physician-Family Caregiver Communication
ITG: Inventory of Traumatic Grief
